# Supplementary material for: Multibandgap quantum dot ensembles for solar-matched infrared energy harvesting
Source: Nat Commun. 2018 Oct 1;9:4003. doi: 10.1038/s41467-018-06342-7 (PMC6167381; doi:10.1038/s41467-018-06342-7)
Supplement: Supplementary file 1 — Supplementary Information [file 41467_2018_6342_MOESM1_ESM.pdf]

Multibandgap Quantum Dot Ensembles for Solar-Matched Infrared Energy Harvesting

- Supplementary Information -

Bin Sun[1^], Olivier Ouellette[1^], F. Pelayo García de Arquer[1^], Oleksandr Voznyy[1], Younghoon Kim[1], Mingyang Wei[1], Andrew H. Proppe[1,2,3], Makhsud I. Saidaminov[1], Jixian Xu[1], Mengxia Liu[1], Peicheng Li[4], James Z. Fan[1], Jea Woong Jo[1], Hairen Tan[1], Furui Tan[1], Sjoerd Hoogland[1], Zheng Hong Lu[4], Shana O. Kelley[2,3], Edward H. Sargent[1]*

[1]Department of Electrical and Computer Engineering, University of Toronto, 10 King's College Road, Toronto, Ontario, M5S 3G4, Canada

[2]Department of Pharmaceutical Science, Leslie Dan Faculty of Pharmacy, University of Toronto, Toronto, ON, M5S 3G4, Canada.

[3]Department of Biochemistry, Faculty of Medicine, University of Toronto, Toronto, ON, M5S 3M2, Canada

[4]Department of Material Science and Engineering, University of Toronto, 184 College St, Toronto, Ontario, M5S 3E4, Canada

*E-mail: ted.sargent@utoronto.ca

^These authors contributed equally to this work.

Supplementary Figures

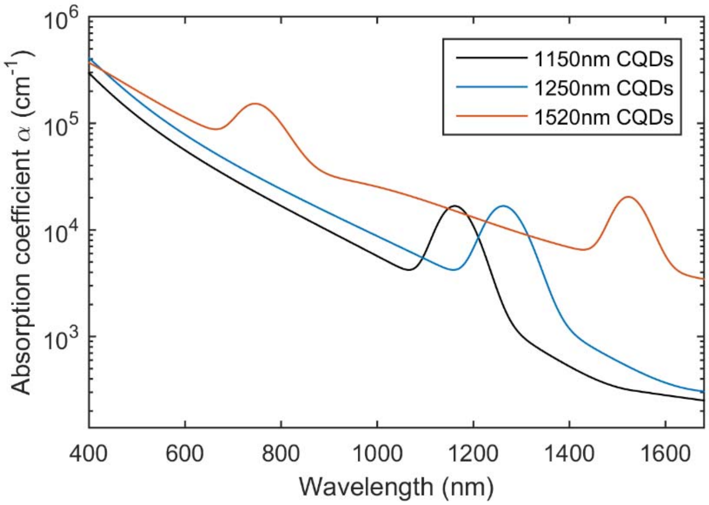

Supplementary Figure 1. Absorption coefficient of the CQD films used to calculate *G*, obtained from spectroscopic ellipsometry.

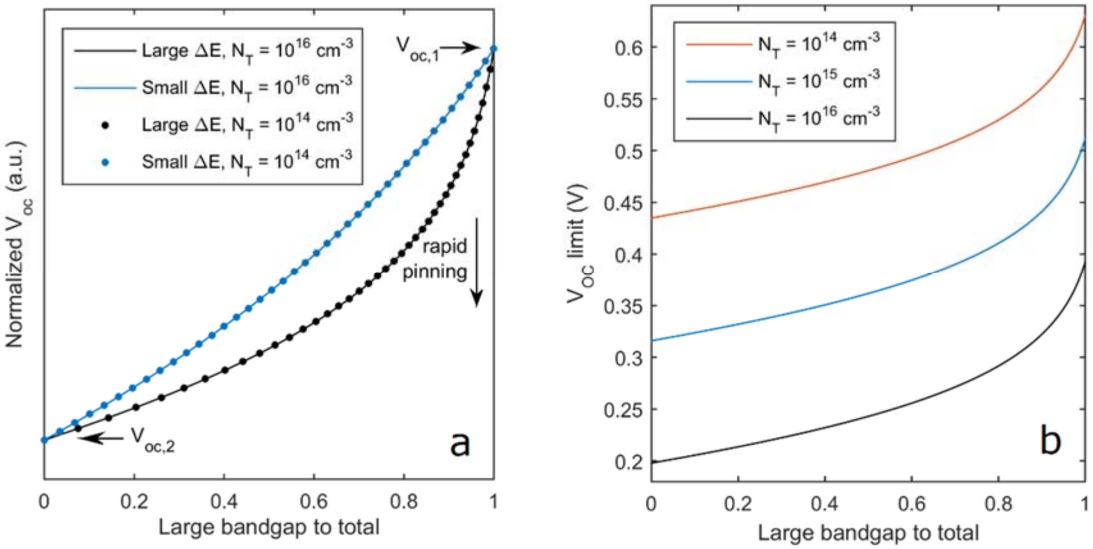

Supplementary Figure 2. Effect of trap density on the V_{OC} model. | a) Calculation done with two different trap densities, illustrating how the V_{OC} pinning trend from Figure 1c is not affected. b) V_{OC} limit in the large *ΔE* case for different trap densities in absolute units, showing that only the magnitude of V_{OC} is changed.

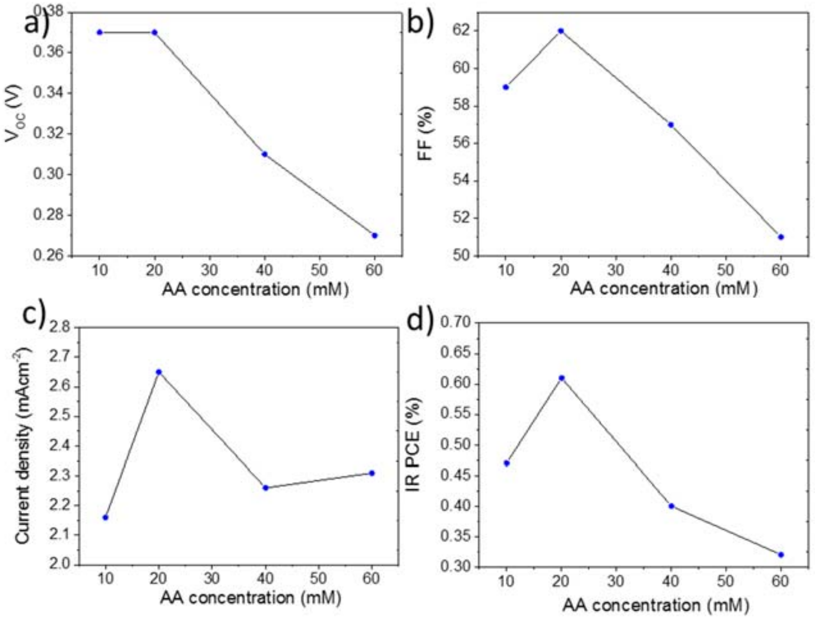

Supplementary Figure 3. Optimization of ligand exchange for large bandgap CQDs. | The optimized AA concentration in the precursor solution is 2 mM in DMF. When the AA concentration is lower than 2 mM, FF and J_{SC} decrease, which is attributed to the high amount of organic ligand on the surface, resulting in worse charge transport. When increasing the AA concentration, surface passivation gets worse, resulting in decreased PV performance, particularly lowering V_{OC} and FF as shown in Supplementary Figure 4a, b.

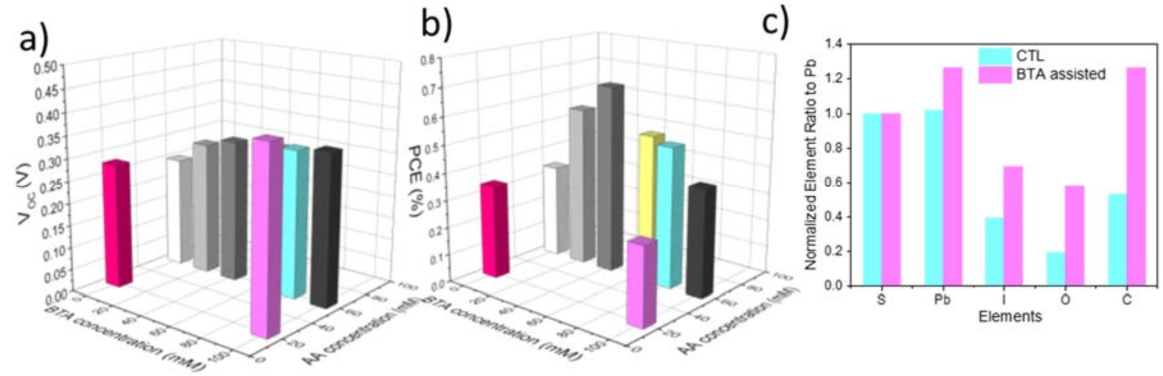

Supplementary Figure 4. BTA assisted ligand exchange on small bandgap dots. | a) & b) Device performance as a function of AA and BTA concentration: V_{OC} increases with increasing BTA concentration and decreases with increasing AA fraction; the highest PCE (0.62%) is obtained when AA (60 mM) and BTA (40 mM) are added, which is higher than the previously reported PCE of solution exchanged 1250 nm PbS CQDs[34]; c) XPS elemental ratios reveal the higher ratio of I:S and C:S when using BTA (40 mM in precursor solution) compared to the control ligand exchange without BTA, showing that the addition of BTA keeps more iodide and organics (Oleic acid) on the CQD surface for better surface passivation.

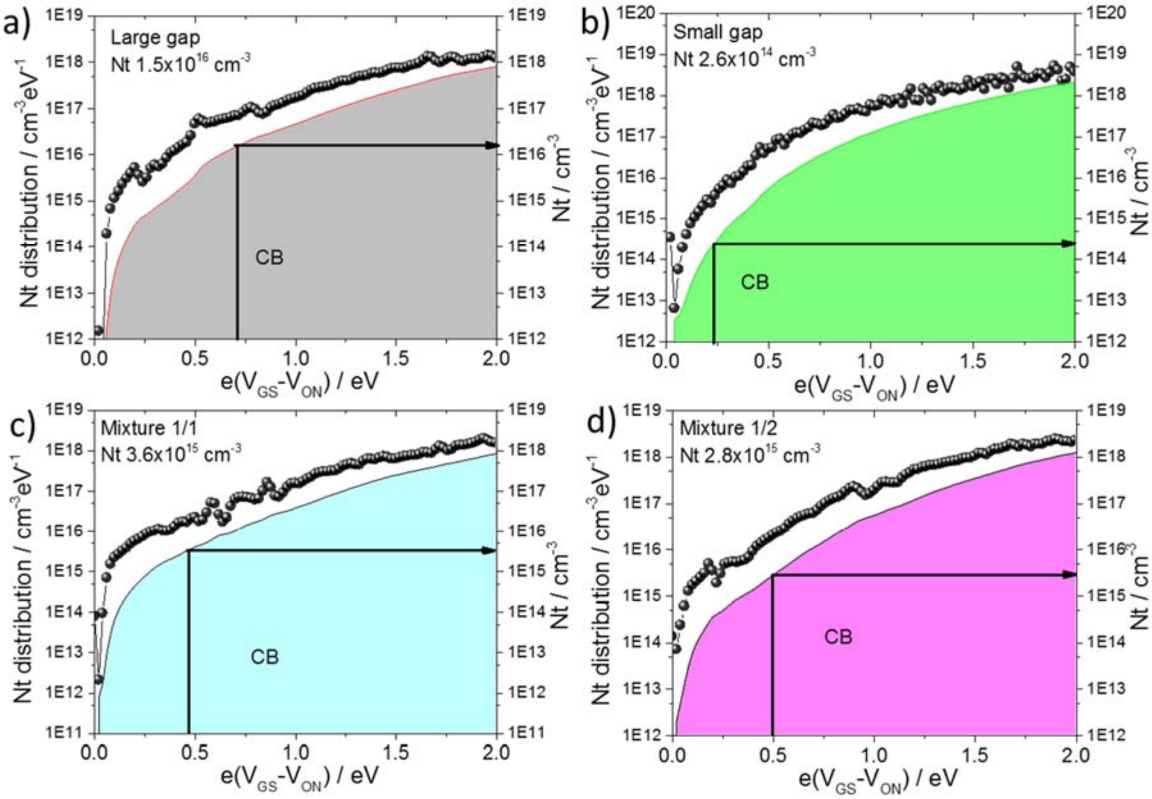

Supplementary Figure 5. Trap density extracted from FET devices. a) single large bandgap CQDs, b) single small bandgap CQDs, c) mixes with 50% of large bandgap CQDs, d) mixes with 33% of large bandgap CQDs.

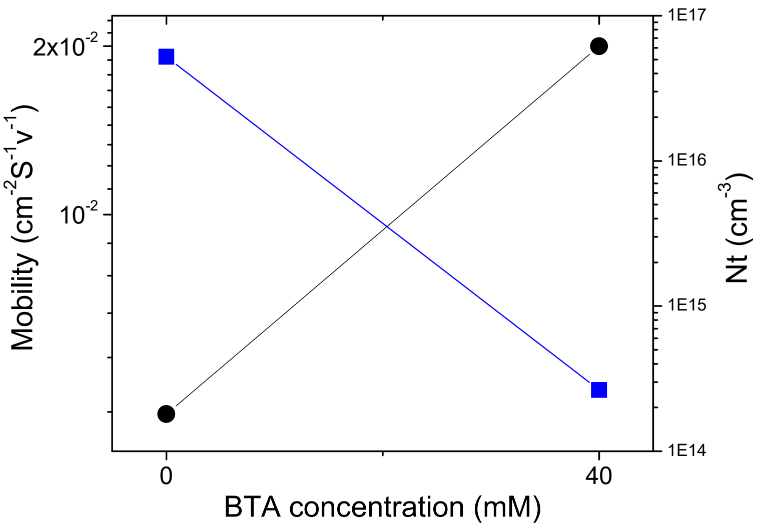

Supplementary Figure 6. Transport properties of small bandgap dots exchanged with and without BTA to assist exchange. | Exchange was done using 60 mM of AA and 40 mM of BTA (if used). Black circles denote the carrier mobility in CQDs. The absence of BTA leads to an electron mobility in the CQD films of 0.0044 $cm^{-2}s^{-1}V^{-1}$, which is one order of magnitude lower than with BTA. The lower mobility is attributed to the surface trap density, which was calculated to be 5.2×10^{16} cm^{-3}, while BTA-assisted films has a much lower surface trap density of 2.6×10^{14} cm^{-3}, in good agreement with the PV device performance shown in Supplementary Figure 4.

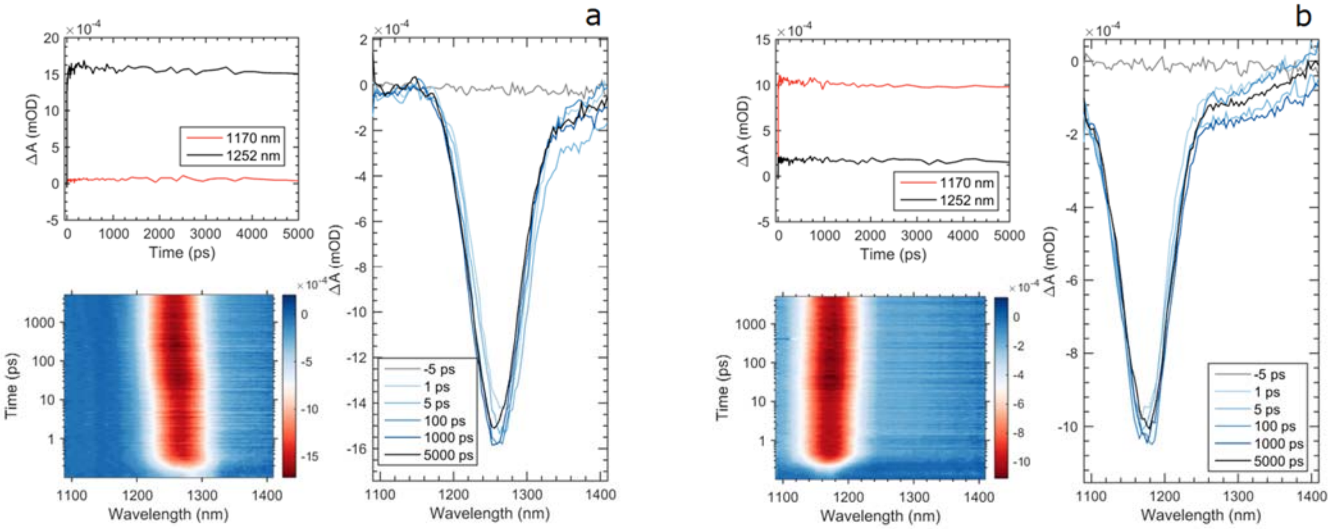

Supplementary Figure 7. Transient absorption spectra of pure CQD films. | Bottom left: 2D spectrum. Top left: Temporal cross-section. Right: Spectral cross-section. (a) Pure small bandgap CQD film, photoexcitation at 1300 nm. (b) Pure large bandgap CQD film, photoexcitation at 1160 nm. The 1300 nm photoexcitation wavelength was chosen to minimize the partial excitation of the small-bandgap population in the mixed CQD sample (Supplementary Figure 8) and was used for the single-size sample for consistency. When studying the pure-phase small-gap film as a control, the absorption change at the wavelength corresponding to the large-gap-phase's excitonic feature is at least 20 times lower than the absorption change at the wavelength corresponding to the small-gap-phase's excitonic feature; conversely, when studying the pure-phase large-gap film as control, the absorption change at the wavelength corresponding to the small-gap-phase's excitonic feature is about 5 times lower than the absorption change at the wavelength corresponding to the large-gap-phase's excitonic feature.

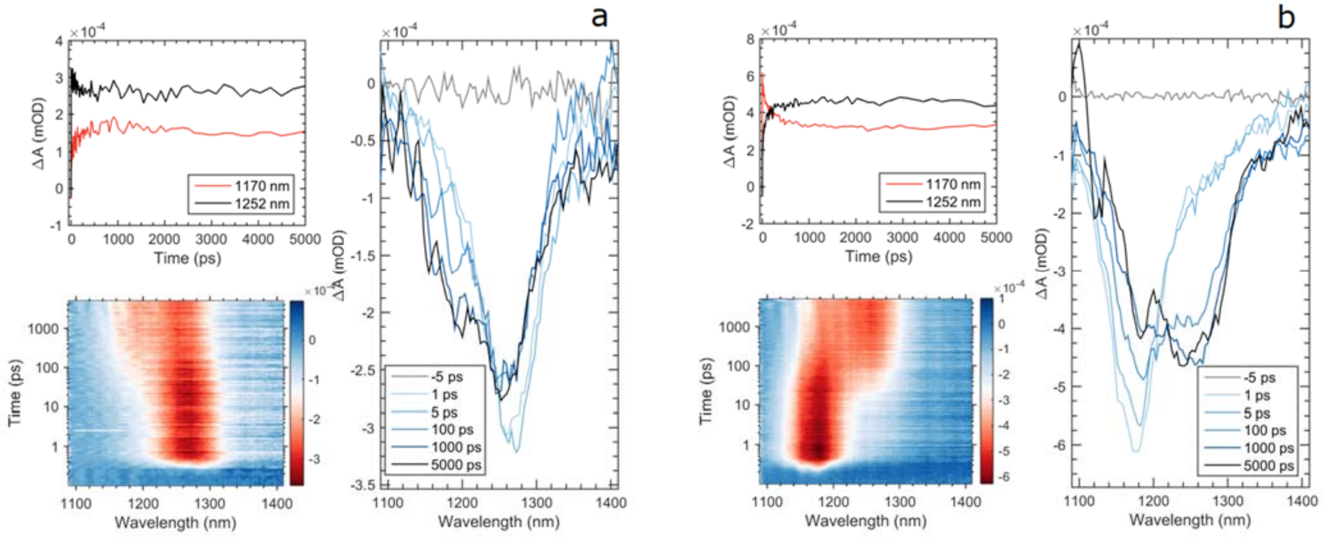

Supplementary Figure 8. Transient absorption spectra of L/S 2/1 mixed CQD films. | Bottom left: 2D spectrum. Top left: Temporal cross-section. Right: Spectral cross-section. (a) Photoexcitation mainly in the small bandgap population at 1300 nm. Although we directly photoexcited very selectively only the small-gap phase (see Supplementary Figure 7a), the bleach at 1170 nm – characteristic of the large-gap phase – is within a factor of two of the bleach at 1265 nm. We conclude that charge carriers are able to transfer mildly-uphill from their place of creation (small-gap phase) into the larger-gap phase. (b) Photoexcitation mainly in the large bandgap population at 1160 nm; the absorption cross-section is approximately 13 times greater in the large-bandgap QDs than in the small-bandgap QDs for the case of 1160 nm photoexcitation. Charge transfer from the photoexcited population to the other is observed in both cases, confirming that thermalization happens before most recombination. Exponential fits to the temporal cross-sections reveal transfer times of 90 ps from large-bandgap to small-bandgap dots and 175 ps from small-bandgap to large-bandgap dots.

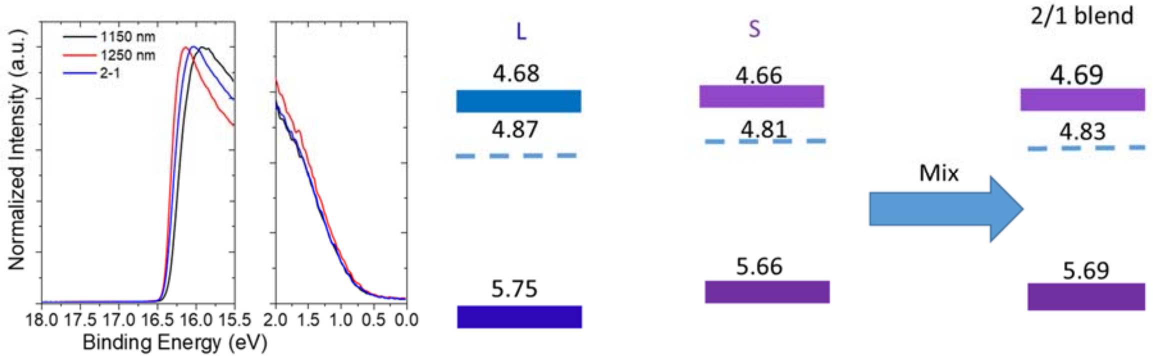

Supplementary Figure 9. Energy levels of CQD films from ultraviolet photoelectron spectroscopy (UPS). | UPS spectra of L, S, and 2-to-1 mixed dots (left) and energy levels (Fermi level (E_F) and valence band (VB)) calculated from UPS spectra. A helium discharge source (HeI α, hv =21.22 eV) was used and the samples were kept at a take-off angle of 88°. During measurement, the sample was held at a −15 V bias relative to the spectrometer in order to efficiently collect low kinetic-energy electrons. E_F was calculated from the equation: E_F = 21.22 eV − SEC, where SEC is the secondary electron cut-off. The difference between valence band (VB) and Fermi level, η, was determined from the VB onset in the VB region. The 1150 and 1250 nm CQDs show very similar E_F and VB maxima, matching well with the energy alignment for charge transport between different size CQDs. The conduction band (CB) is extracted from the absorption spectra using the position of the first exciton peak.

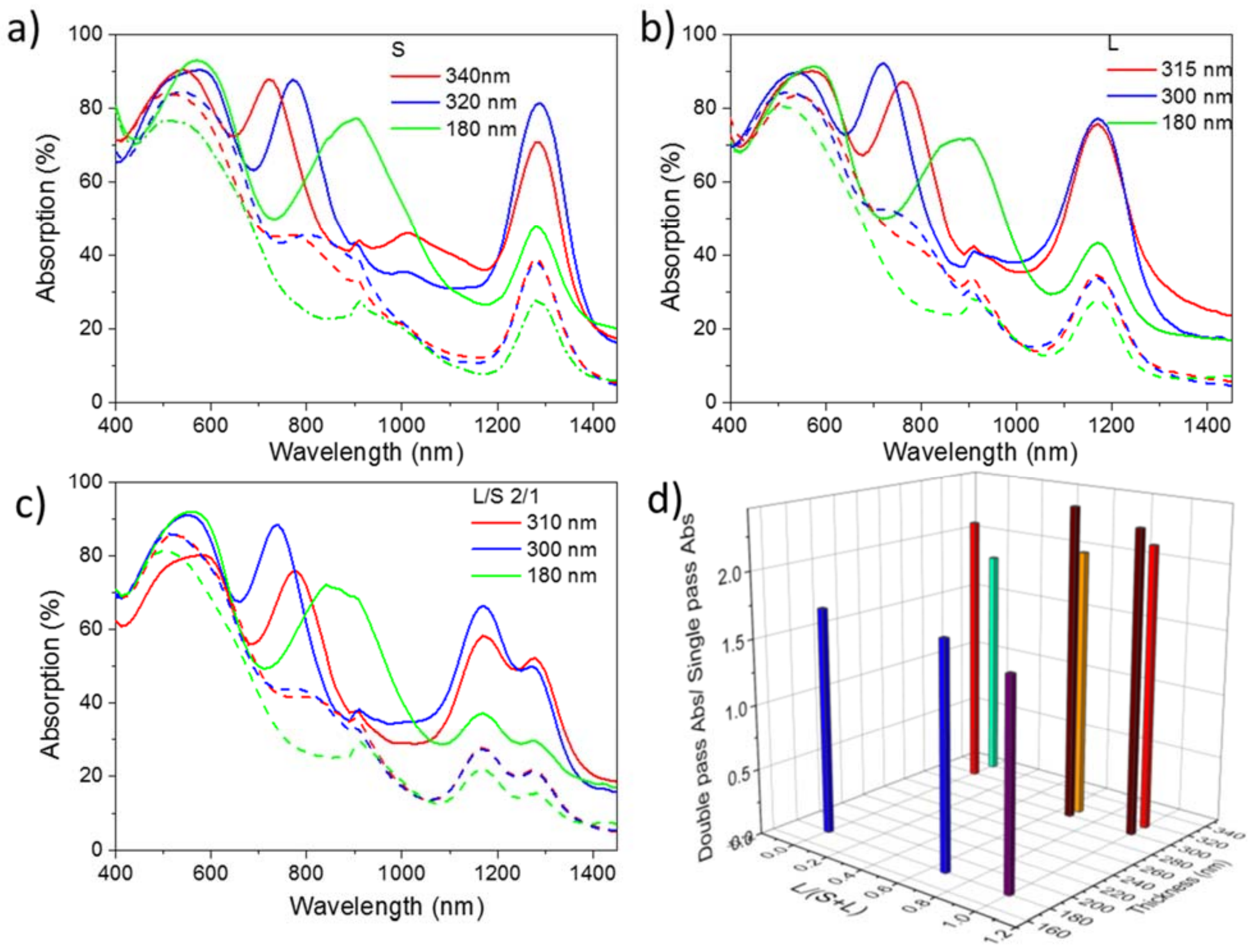

Supplementary Figure 10. Resonant enhanced light absorption. Absorptance measured from double pass (solid line, with gold electrode mirror) and single pass (dashed line, without gold electrode mirror) of solar cells with different CQD active layer thicknesses. a) small bandgap CQD film; b) large bandgap film; c) mixture containing 67% of large bandgap CQDs; d) ratio of double pass over single pass absorption at the position of the highest exciton peak; the ratio increases with thickness from 180 nm to 300 nm, then decreases when the thickness is further increased.

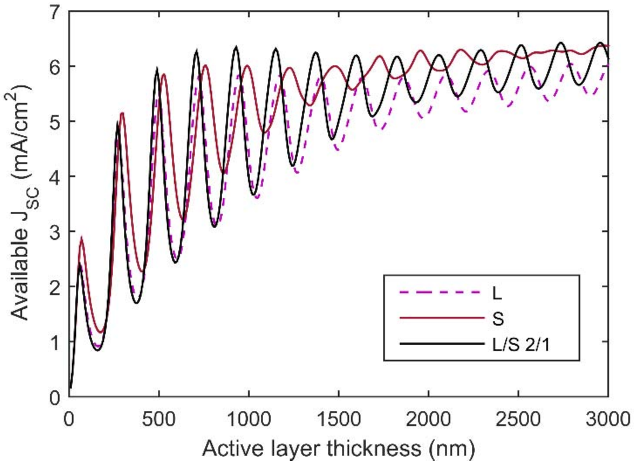

Supplementary Figure 11. Available J_{SC} in thick active layers, illustrating the role of optical resonance in enhancing light absorption.

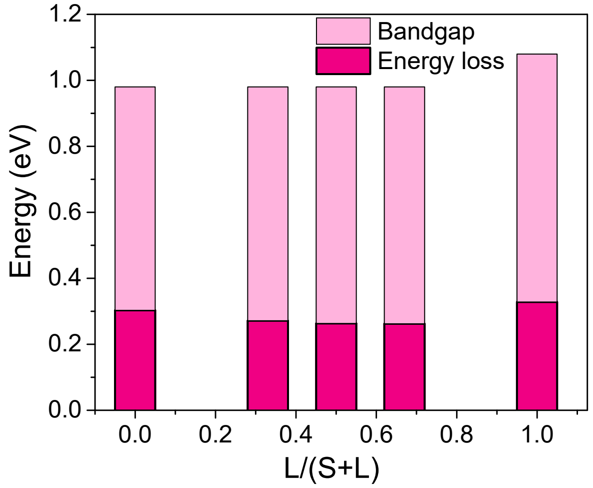

Supplementary Figure 12. Energy loss dependence on the inclusion of large bandgap CQDs in mixed CQD films under full AM1.5G irradiation. The large bandgap CQDs have the largest E_{loss} of 0.33 eV, while the small bandgap CQDs show an E_{loss} 0.30 eV. After mixing, the 2-to-1 and 1-to-1 mixed CQD films both show an E_{loss} of 0.26 eV, and the 1-to-2 mixed CQD film has slightly higher E_{loss} of 0.27 eV, all of which are much lower than single CQD films.

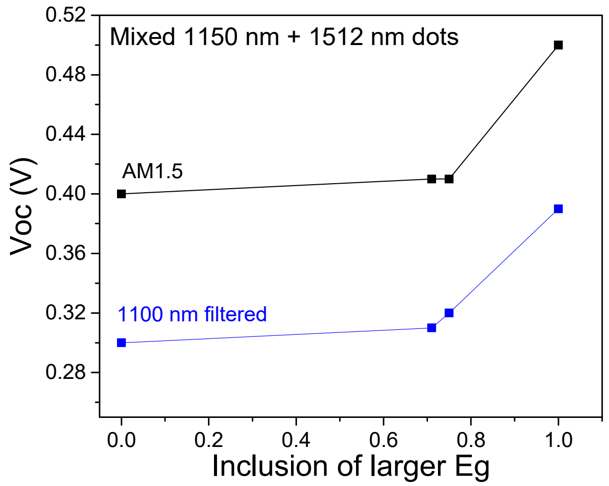

Supplementary Figure 13. V_{OC} change versus inclusion of large gap CQDs in mixtures of 1150 nm and 1512 nm. | The V_{OC} of mixtures is pinned to that of the low bandgap CQDs, when the bandgap difference is 0.26 eV. This fast pinning is in good agreement with the theoretical model.

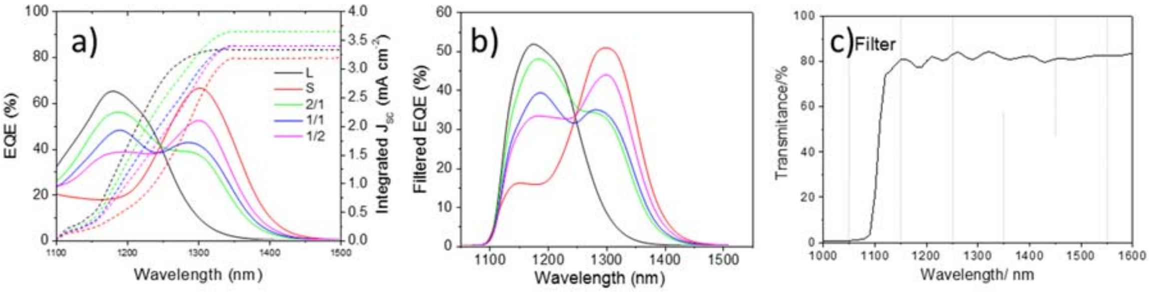

Supplementary Figure 14. EQE curves and expected J_{SC} integrated under AM1.5G irradiation. | EQE curves without (a) and through (b) 1100 nm long-pass filter; transmittance of the 1100 nm long-pass filter used in this work.

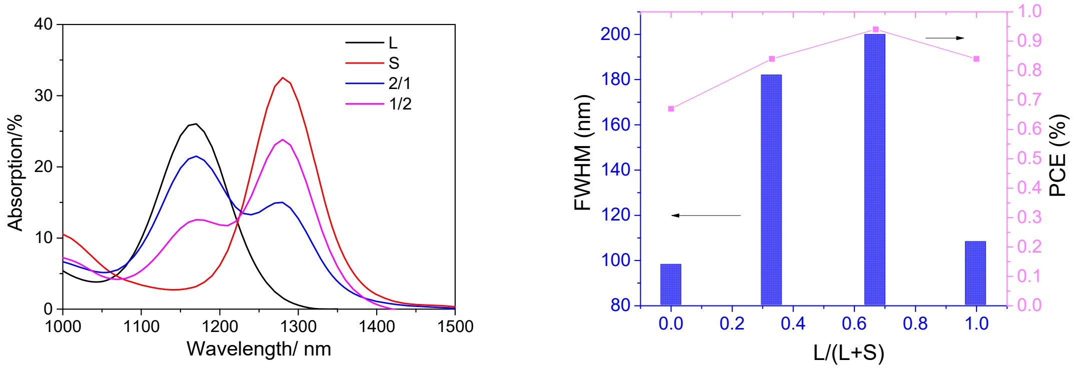

Supplementary Figure 15. Impact of exciton peak width | Full width at half max (FWHM) (right) extracted from the absorption spectrum of single size CQDs and mixes (left). The mixed CQD films exhibit a larger FWHM, which contributes to their higher J_{SC} and PCE.

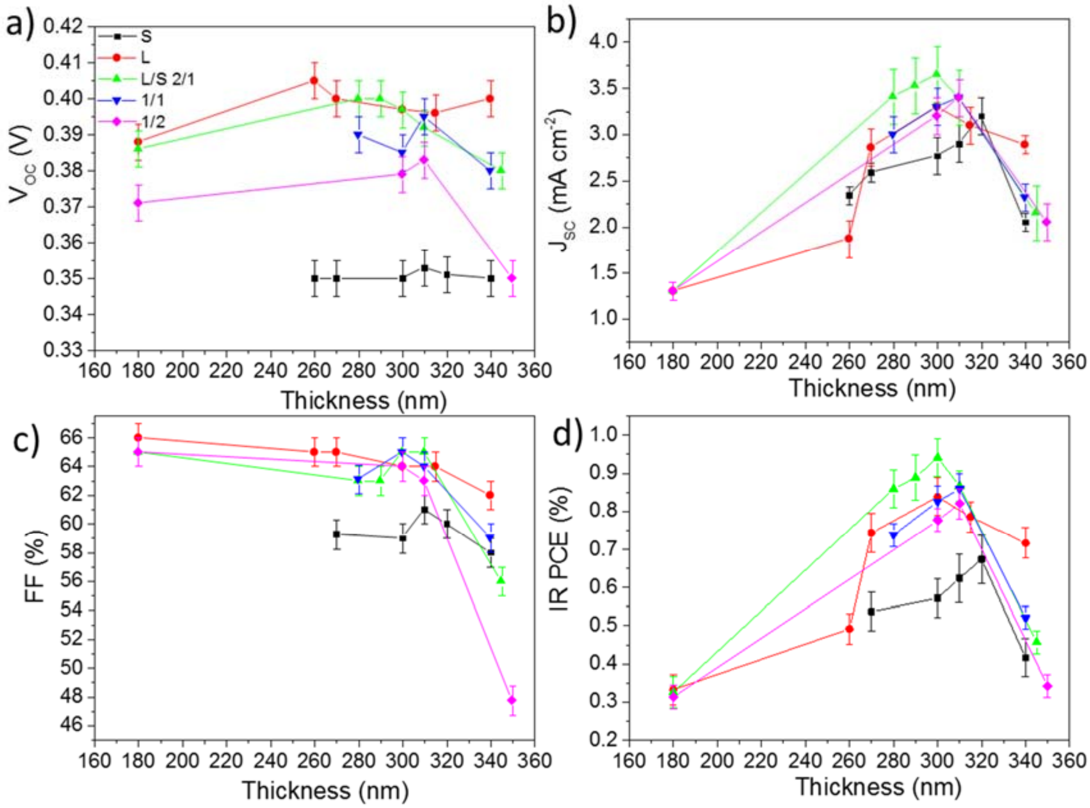

Supplementary Figure 16. Thickness dependence of the IR PV performance. | a) V_{OC}, b) J_{SC}, c) FF, d) IR PCE of large bandgap film, small bandgap CQD film, mixture containing 67%, 50%, and 33% of large bandgap CQDs.

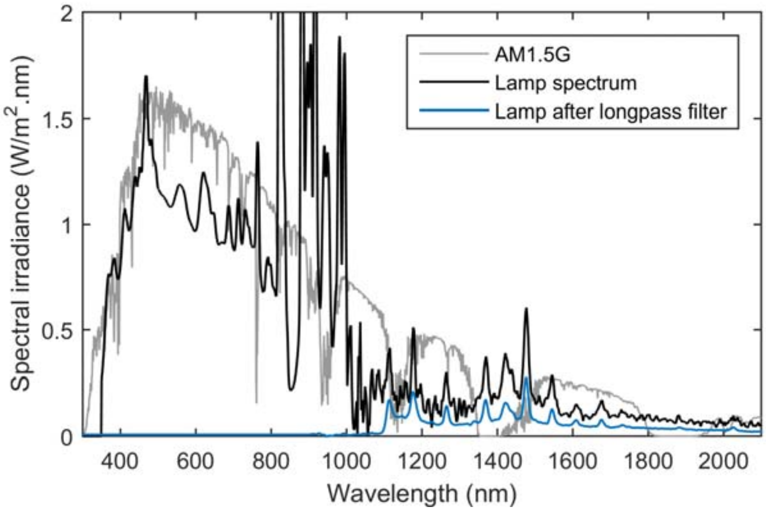

Supplementary Figure 17. Solar simulator lamp spectrum, with and without 1100 nm long-pass filter, with the AM1.5G standard spectrum for comparison.

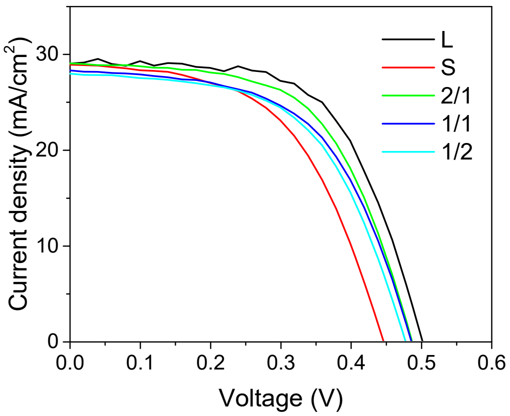

Supplementary Figure 18. *J-V* characteristics of single size CQDs and mixes under AM1.5G.

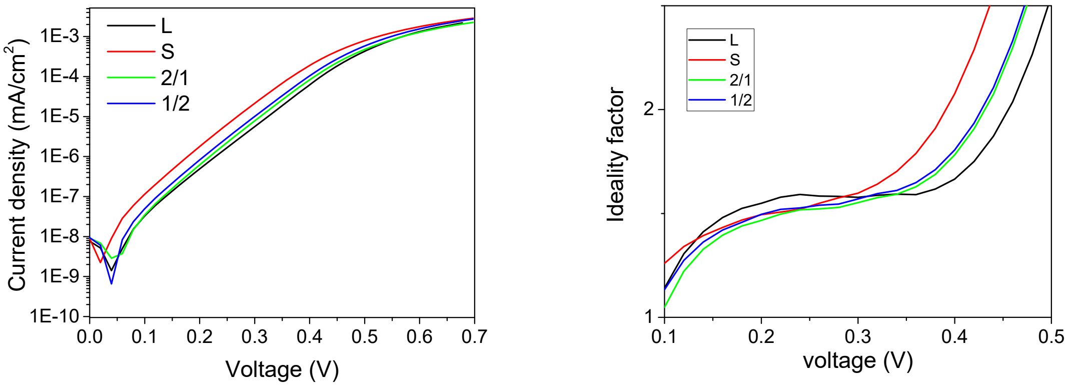

Supplementary Figure 19. Dark diode analysis of best PV device with different inclusion of large bandgap CQDs. | a) dark *IV* and b) instantaneous ideality factor extracted from dark *IV*. In the quasi-flat region, the ideality factor of small bandgap CQDs and mixed films is slightly lower than that of the large bandgap CQD films. This is an indication of a higher density of tail states in large bandgap CQDs, in good agreement with the FET data. The ideality factor increasing above 2 at higher voltages is due to series resistance.

Supplementary Tables

Supplementary Table 1: Numerical values used in V_{OC} calculation.

		Small ΔE	Large ΔE
QD bandgap	E_{exc}	1.08 eV, 1.00 eV (1150 nm, 1250 nm)	1.08 eV, 0.82 eV (1150 nm, 1520 nm)
QD diameter	d	3.9 nm, 4.4 nm	3.9 nm, 5.7 nm
QD size distribution standard deviation	σ	40 meV (4% size dispersity)	40 meV (4% size dispersity)
Temperature	T	300 K	300 K
QD packing density	P	0.65	0.65
Lowest-energy excited state degeneracy	δ	8	8
Trap density	N_T	10^{16} cm^{-3}	10^{16} cm^{-3}
Charge mobility	μ	0.02 cm^{2}V^{-1}s^{-1}	0.02 cm^{2}V^{-1}s^{-1}
Excited carrier lifetime	τ	480 ns, 435 ns	480 ns, 330 ns
Photogeneration rate	G	3.7×10^{20} cm^{-3}s^{-1}, 5.2×10^{20} cm^{-3}s^{-1}	3.7×10^{20} cm^{-3}s^{-1}, 1.1×10^{21} cm^{-3}s^{-1}

Supplementary Table 2. Performance summary of optimal solar cells under AM1.5 irradiation and IR performance >1100 nm at optimal thickness from more than 10 devices.

	Large gap CQD fraction	0	33%	50%	67%	100%
AM1.5G performance at optimal thickness	Thickness	320 nm	300 nm	300 nm	300 nm	300 nm
	V_{OC} (V)	0.45±0.005	0.47±0.005	0.48±0.005	0.49±0.005	0.50 ±0.005
	J_{SC} (mA cm^{-2})	29±0.5	28±0.5	28.3±0.5	29.4±0.5	29±0.5
	FF(%)	54±1	60±1	61±1	59±1	61±1
	PCE (%)	7.0±0.3	8.0±0.3	8.3±0.3	8.5±0.3	8.9±0.2
IR performance at optimal thickness	Thickness	320 nm	310 nm	310 nm	300 nm	300nm
	V_{OC} (V)	0.35±0.005	0.38±0.005	0.39±0.005	0.40±0.005	0.40 ±0.005
	J_{SC} (mA cm^{-2})	3.2±0.2	3.4±0.2	3.4±0.2	3.7±0.3	3.3±0.2
	FF(%)	60±1	63±1	64±1	65±1	64±1
	PCE (%)	0.67±0.06	0.82±0.04	0.86±0.04	0.94±0.05	0.84±0.05

Supplementary Table 3. Spectral mismatch factor calculated from the EQE spectrum of each device.

Device	Spectral mismatch factor
S	2.04
L	1.82
L/S 2/1	1.86
L/S 1/1	1.8
L/S 1/2	1.83

Supplementary Notes

Supplementary Note 1: V_{OC} calculation details and parameters

The calculation of V_{OC} is based on the detailed balance procedure as described in [1]. When setting the photoexcited charge carrier generation rate G equal to the recombination rate through mid-gap trap states, one can obtain the following equation:

$$G = \frac{n_\mathrm{i}\{\exp[(\varepsilon_\mathrm{FC} - \varepsilon_\mathrm{FV})/kT] - 1\}}{\tau_\mathrm{h,min}\{\exp[(\varepsilon_\mathrm{FC} - \varepsilon_\mathrm{i})/kT] + \exp[(\varepsilon_\mathrm{imp} - \varepsilon_\mathrm{i})/kT]\} + \tau_\mathrm{e,min}\{\exp[(\varepsilon_\mathrm{i} - \varepsilon_\mathrm{FV})/kT] + \exp[(\varepsilon_\mathrm{i} - \varepsilon_\mathrm{imp})/kT]\}}, \quad (1)$$

where n_i is the intrinsic carrier density, ε_{FC} and ε_{FV} are the electron and hole quasi-fermi levels in the conduction and valence band, k is Boltzmann's constant, T is temperature, $\tau_\mathrm{h,min}$ and $\tau_\mathrm{e,min}$ are the minimum hole and electron lifetime, ε_i is the intrinsic fermi level and ε_imp, the trap energy level. Assuming symmetric properties for holes and electrons for simplicity, this expression reduces to

$$G = \frac{n_\mathrm{i}\{\exp[(\varepsilon_\mathrm{FC} - \varepsilon_\mathrm{FV})/kT] - 1\}}{2\tau_\mathrm{min}\{\exp[(\varepsilon_\mathrm{FC} - \varepsilon_\mathrm{FV})/2kT] + \cosh[(\varepsilon_\mathrm{imp} - \varepsilon_\mathrm{i})/kT]\}}, \quad (2)$$

which reduces further in the case of mid-gap traps ($\varepsilon_\mathrm{imp} = \varepsilon_\mathrm{i}$) to

$$G = \frac{n_\mathrm{i}\{\exp[(\varepsilon_\mathrm{FC} - \varepsilon_\mathrm{FV})/kT] - 1\}}{2\tau_\mathrm{min}\{\exp[(\varepsilon_\mathrm{FC} - \varepsilon_\mathrm{FV})/2kT] + 1\}} \approx \frac{n_\mathrm{i}\{\exp[(\varepsilon_\mathrm{FC} - \varepsilon_\mathrm{FV})/2kT]\}}{2\tau_\mathrm{min}}. \quad (3)$$

Knowing all other parameters, this can then be numerically solved to find the quasi-fermi level splitting, $\varepsilon_\mathrm{FC} - \varepsilon_\mathrm{FV}$.

The carrier lifetime τ is calculated from the trap density N_T, thermal velocity v_th and capture cross-section s, as

$$\tau = \frac{1}{N_\mathrm{T} v_\mathrm{th} s}, \quad (4)$$

where s is approximated as the cross-section of a quantum dot and v_th, defined in the hopping regime as d/τ_hop, is obtained from the mobility[2]:

$$v_\mathrm{th} = \frac{6kT\mu}{d}. \quad (5)$$

The carrier generation rate G is calculated from the absorption coefficient $\alpha(\lambda)$ and the incident photon flux $\gamma(\lambda)$ (corresponding to the IR-filtered AM1.5G solar spectrum divided by hc/λ):

$$G = \int_{1100\mathrm{nm}}^{\infty} \alpha(\lambda)\gamma(\lambda)\mathrm{d}\lambda. \quad (6)$$

The absorption coefficients $\alpha(\lambda)$ used in the calculation are shown in Supplementary Figure S1.

To calculate n_i, we first build the conduction band DOS, $g_{CB}(E)$:

$$g_{CB}(E) \approx \frac{\delta P}{V_{exc}} \frac{1}{\sqrt{2\pi\sigma^2}} \exp\left(-\frac{(E-E_{exc})^2}{2\sigma^2}\right), (7)$$

where δ is the degeneracy of the lowest energy state, P is the dot packing density, V_{exc} is the average volume of a dot, E_{exc} is the average lowest energy state (equal to the first excitonic peak position in the absorption spectrum) and σ is the standard deviation of the distribution. V_{exc} is calculated by approximating the dots as spheres. The central position and FWHM of the exciton peak in the CQD films absorption spectra were used to extract the parameters of the gaussian distribution. Assuming the fermi level lies approximately in the middle of the bandgap, n_i can then be evaluated:

$$n_i = \int_{E_C}^{\infty} f(E) g_{CB}(E) dE , (8)$$

where $f(E)$ is the Fermi-Dirac distribution. Finally, the QD diameter *d* is obtained from equation (2) given in the main text.

In the case of a mix of two CQD populations with a different mean size and mixing proportion *x*, the effective DOS is estimated to be a weighted sum of both populations' DOS:

$$g_{CB,total}(E) = x\, g_{CB,1} + (1-x) g_{CB,2} . (9)$$

The trap density was kept constant in the calculation in order to isolate the effects of CQD mixing only on V_{OC} pinning. Supplementary Figure 2a illustrates that the trend in V_{OC} pinning remains identical for different trap densities, while only the magnitude of V_{OC} is affected, as shown in Supplementary Figure 2b.

The numerical values used in the calculations are given in Supplementary Table 1.

Supplementary References:

1. Peter Würfel, U. W. *Physics of Solar Cells: From Basic Principles to Advanced Concepts, 3rd Edition*. (2016).
2. Guyot-Sionnest, P. Electrical Transport in Colloidal Quantum Dot Films. *J. Phys. Chem. Lett.* **3,** 1169–1175 (2012).
3. Fan, J. Z. *et al.* Halide Re-Shelled Quantum Dot Inks for Infrared Photovoltaics. *ACS Appl. Mater. Interfaces* **9,** 37536–37541 (2017).
4. Kiani, A. *et al.* Single-step colloidal quantum dot films for infrared solar harvesting. *Appl. Phys. Lett.* **109,** (2016).
